# The Danger of Large Doses of Quinine

**Published:** 1884-03

**Authors:** A. A. Smith

**Affiliations:** Professor of Materia Medica and Therapeutics, and of Clinical Medicine in Bellevue Hospital Medical College


### THE DANGER OF LARGE DOSES OF QUININE.*

BY A. A. SMITH, M.D.,

*Professor of Materia Medica and Therapeutics, and of Clinical Medicine in Bellevue Hospital Medical College.*

[New York Medical Journal.]

Is the use of quinine in large doses ever dangerous in hyperpyrexia with tendency to heart failure?

In order that I may not be charged with prejudice in regard to the use of quinine, let me say at the outset: I place it second in importance only to opium, in our armamentarium of drugs, in the treatment of disease in this region.

It will be admitted that one of the most serious dangers to be apprehended in any disease in which high temperature prevails for a few days is the so-called heart failure. Many observers contend that this heart failure is due to the direct effect of the great heat on the heart muscular tissue, producing in many cases a permanent degeneration, in others a temporary enfeeblement. Other observers consider it due to depression of nerve force, and that no degeneration of heart muscular tissue occurs. It is possible both views are correct. In some cases it may be through the nervous system; in others, and it seems to me the more rare condition, muscular degeneration may occur. If real muscular degeneration does occur, there ought to remain, even if the patient recovers from his attack of fever, permanent enfeeblement of the heart. Whether through the one system or the other, it is of serious import to the patient, for in many cases death is directly traceable to this heart failure.

As the two types of fever, typhoid may represent the fevers of long duration, and pneumonia those of short duration.

During the first few days, usually, of a typical acute lobar pneumonia, there is an increased rapidity of pulse; it is full and bounding and non-compressible; the first sound of the heart is intensified; there is some cerebral excitement, probably due, in a measure, to the increased vascular excitement. After the first three days, the pulse in severe cases increases in rapidity, but usually diminishes in force, and the first sound of the heart is diminished in intensity. These signs are usually regarded as evidences of exhaustion. The fatal result occurs in many cases on the fourth, fifth or sixth day. From the third to the sixth day may be regarded as the dangerous

*Read before the New York Clinical Society, November 23, 1883.

days, as the rule. In typhoid fever, the disease goes on developing up to the sixth, to the tenth day, and then there begin those evidences of heart failure as shown by the increased rapidity of pulse and diminution in its force, and decreasing intensity of the first sound of the heart. These descriptions give a general idea of the progress of these two diseases so far as the heart is concerned. There are, of course, many exceptions to the rule as stated above. Cases do occur with the progress as here stated, and will answer our purpose in the discussion of the question.

Almost all observers agree that when quinine is given as an antipyretic it should be given in one single large dose, or at least a large quantity must be given within an hour. Usually this need not be repeated within twenty-four hours. Others give the large doses at twelve hours' interval. A large dose may be anywhere from twenty to sixty grains, depending somewhat on the observer and somewhat on the height to which the temperature has risen.

The physiological effect of quinine on the circulatory system is a subject of great interest, and especially so to us to-night, in view of the question for discussion.

Almost all observers agree that small doses of quinine, say from two to five grains, stimulate the heart both as to frequency and force.

Almost all observers agree that large doses act, in many cases, as powerful antipyretics. Some even attempt to explain their antipyretic effects by their influence as an arterial and cardiac sedative. I will not enter into any discussion as to how quinine acts as an antipyretic; its effect on the heart, directly or indirectly, is what concerns us in the discussion to-night. The effects of quinine on the circulation may be studied from two standpoints—that of the experimentalist and that of the clinician. I admit that many experiments may lose much of their value from the fact that the observer makes his experiments under the influence of a very strong dominant idea. Therefore great care should be shown in estimating the value of any experiments.

The experiments of Eulenburg and other observers seem to show that quinine in sufficient quantity causes a diastolic cardiac arrest by a direct depressant action on the heart. Other experiments seem to show that toxic doses of quinine paralyze the vaso-motor nervous system.

Binz's experiments seem to show that some of the disturbances of sight and hearing depend on impairment of the heart's function. He thinks experiments on warm-blooded animals have proved that the injury to the heart and paralysis of the respiratory center are the cause of death.

Bartholow thinks that quinine in large doses depresses the action of the heart, diminishes the blood-pressure, and enfeebles while it slows the pulse.

Farquharson says large doses of quinine cause the rate of the pulsation to fall and the arterial tension to diminish, and adds that death may even ensue from convulsions or sudden collapse following depression of the heart's action.

H. C Wood, Jr., says: "Quinine, when given in large doses, acts as a powerful depressant, and for this reason is given in some diseases where such action is desired." Many observers have noticed that large doses (some specifying from thirty to sixty grains) produced in man a less frequency and less force of the pulse. Sometimes after very large doses it has been observed that the radial pulse was almost imperceptible. When it became as feeble as this, it was rapid and sometimes irregular. I have frequently observed, even after twenty grains of quinine, that, although while the patient was in a recumbent posture very little change in the frequency of the pulse was noticed, yet when the patient got up and walked about, even leisurely, there was decided increase in the rapidity of the pulse, and some diminishing of its force.

The pallor of countenance, with coolness of lips and skin and coldness of extremities, which sometimes follows large doses of quinine, seem to me to be due to depression of the heart's action or a depression of the circulation through the vaso-motor nervous system.

In some patients there is a great susceptibility to the influence of quinine, and frequently "fluttering" at the pit of the stomach and over the præcordial region, with coldness of hands and feet, is complained of.

A gentleman in this city, past sixty years of age, who was formerly in the habit of taking considerable quinine—sometimes as much as thirty grains a day—has told me that after he has taken a quantity above twenty grains a day he has noticed a decided dyspnœa on exercise, fluttering over his præcordial region, a less forcible pulse, and sometimes an irregular one.

I have in a number of instances noticed patients, to whom I have been giving large doses of quinine to reduce temperature, have the temperature suddenly fall, while they passed into a condition of exhaustion with profuse perspiration, feeble pulse, cold hands and feet, and sighing respiration.

I have for some years felt that, if the physiological effects as above described are correct, there comes a time in almost all febrile conditions when quinine *may* be dangerous. This time is when the heart begins to fail, and this usually occurs from the third to the sixth day in pneumonia, and during the second week in typhoid fever, taking these again merely as types of fevers of short and long duration. The temperature after heart failure begins may be as high as, or even higher than before, certainly a very important fact in view of the foregoing statements, and in view of the almost routine practice of prescribing large doses of quinine in high temperature, whatever the condition of the heart may be.

Large doses of quinine begin their effects in from a half hour to two hours. They reach their maximum effects in from four to six hours. Agents can be given to counteract the effects of large doses of quinine on the heart, and should always be given where there is the tendency to heart failure. When they are given, their effects should be kept up for many hours. The effects of some of the cardiac stimulants pass off in a short time. The remedy must be given in such a way that its effects will be kept up continuously. If a single large dose of quinine is given, it would be practically useless, for instance, to give a single dose of ammonia to counteract the distressing effects of the quinine. It must be given in doses repeated every two or three hours, and kept up even after the effects of the quinine have passed off. Almost any of the cardiac stimulants will aid in counteracting the effects of these large doses of quinine; but I have found small doses of opium answer the best purpose. In doses from a quarter to a half grain, opium is a cardiac stimulant, besides being, under conditions of high temperature, a great sedative to the nerve perturbation which accompanies such a condition.

My observations have led me to the following conclusions, although I am still open to conviction:

Large doses of quinine should not be given in any case of high temperature after heart failure begins, unless agents to counteract their effects on the heart are given.

Great caution should be exercised in giving large doses of quinine, in high temperature, in any case of organic heart disease with enfeebled power. They should be used cautiously in old people with high temperatures.

I am well aware of the popular prejudice against quinine, and have hesitated somewhat in calling attention to some of the points involved in this question, because there are so many conditions which can be relieved by quinine better than by any other agent. Indeed, I can only repeat what I said at the beginning of this discussion—I place it second in importance only to opium in the treatment of disease in this region.

